# Self-report underestimates the frequency of the acute respiratory exacerbations of COPD but is associated with BAL neutrophilia and lymphocytosis: an observational study

**DOI:** 10.1186/s12890-024-03239-8

**Published:** 2024-09-02

**Authors:** Yorusaliem Abrham, Siyang Zeng, Wendy Lin, Colin Lo, Alexander Beckert, Laurel Evans, Michelle Dunn, Brian Giang, Krish Thakkar, Julian Roman, Paul D. Blanc, Mehrdad Arjomandi

**Affiliations:** 1 Medical Service, San Francisco Veterans Affairs Health Care System, San Francisco, CA USA; 2 Department of Medicine, University of California, San Francisco, CA USA; 3 Department of Biomedical Informatics and Medical Education, University of Washington, Seattle, USA; 4 Chicago College of Osteopathic Medicine, Midwestern University, Downers Grove, IL USA

**Keywords:** COPD exacerbation, Smoking, Questionnaire, Bronchoalveolar lavage, Airway inflammation, Neutrophils, Lymphocytes

## Abstract

**Rationale:**

Research studies typically quantify acute respiratory exacerbation episodes (AECOPD) among people with chronic obstructive pulmonary disease (COPD) based on self-report elicited by survey questionnaire. However, AECOPD quantification by self-report could be inaccurate, potentially rendering it an imprecise tool for identification of those with exacerbation tendency.

**Objective:**

Determine the agreement between self-reported and health records-documented quantification of AECOPD and their association with airway inflammation.

**Methods:**

We administered a questionnaire to elicit the incidence and severity of respiratory exacerbations in the three years preceding the survey among current or former heavy smokers with or without diagnosis of COPD. We then examined electronic health records (EHR) of those with COPD and those without (tobacco-exposed persons with preserved spirometry or TEPS) to determine whether the documentation of the three-year incidence of moderate to very severe respiratory exacerbations was consistent with self-report using Kappa Interrater statistic. A subgroup of participants also underwent bronchoalveolar lavage (BAL) to quantify their airway inflammatory cells. We further used multivariable regressions analysis to estimate the association between respiratory exacerbations and BAL inflammatory cell composition with adjustment for covariates including age, sex, height, weight, smoking status (current versus former) and burden (pack-years).

**Results:**

Overall, a total of 511 participants completed the questionnaire, from whom 487 had EHR available for review. Among the 222 participants with COPD (70 ± 7 years-old; 96% male; 70 ± 38 pack-years smoking; 42% current smoking), 57 (26%) reported having any moderate to very severe AECOPD (m/s-AECOPD) while 66 (30%) had EHR documentation of m/s-AECOPD. However, 42% of those with EHR-identified m/s-AECOPD had none by self-report, and 33% of those who reported m/s-AECOPD had none by EHR, suggesting only moderate agreement (Cohen’s Kappa = 0.47 ± 0.07; *P* < 0.001). Nevertheless, self-reported and EHR-identified m/s-AECOPD events were both associated with higher BAL neutrophils (ß ± SEM: 3.0 ± 1.1 and 1.3 ± 0.5 per 10% neutrophil increase; *P* ≤ 0.018) and lymphocytes (0.9 ± 0.4 and 0.7 ± 0.3 per 10% lymphocyte increase; *P* ≤ 0.041). Exacerbation by either measure combined was associated with a larger estimated effect (3.7 ± 1.2 and 1.0 ± 0.5 per 10% increase in neutrophils and lymphocytes, respectively) but was not statistically significantly different compared to the self-report only approach. Among the 184 TEPS participants, there were fewer moderate to very severe respiratory exacerbations by self-report (*n* = 15 or 8%) or EHR-documentation (*n* = 9 or 5%), but a similar level of agreement as those with COPD was observed (Cohen’s Kappa = 0.38 ± 0.07; *P* < 0.001).

**Discussion:**

While there is modest agreement between self-reported and EHR-identified m/s-AECOPD, events are missed by relying on either method alone. However, m/s-AECOPD quantified by self-report or health records is associated with BAL neutrophilia and lymphocytosis.

**Supplementary Information:**

The online version contains supplementary material available at 10.1186/s12890-024-03239-8.

## Introduction

Chronic Obstructive Pulmonary Disease (COPD) is projected to be one of the four leading causes of death worldwide by 2030 [1]. Acute respiratory exacerbation of COPD (AECOPD), a rapid worsening from baseline of COPD status to symptoms including dyspnea, cough, and mucus production, is believed to be a major contributor to morbidity and mortality in COPD [2, 3]. In addition, many people at risk for COPD due to smoking but without a formal clinical diagnosis including spirometric confirmation (tobacco-exposed persons with preserved spirometry or TEPS) also experience respiratory exacerbations consistent with AECOPD [4, 5]. Many clinical research studies of COPD management employ AECOPD frequency and severity as the end-points of interest [6–8]. Additionally, clinicians may use AECOPD frequency and severity to determine the appropriate clinical management and treatment of COPD [9]. To assess the patients’ history of AECOPD, researchers and clinicians typically rely on self-reported questionnaire items to capture the frequency and severity of AECOPD [10, 11]. Self-report, however, may be susceptible to recall bias as well as confusion between what may have been another acute or chronic illness besides smoking and COPD causing respiratory problems [12–15].

In this study, we aimed to determine whether self-report based on a self-completed COPD-relevant respiratory exacerbation battery is valid in quantifying the frequency and severity of AECOPD. We hypothesized that defining AECOPD solely on self-report leads to inaccuracies, including under- and over-reporting. To test this, we administered a respiratory exacerbation/AECOPD questionnaire to persons with a history of heavy smoking, not all of whom carried a known COPD diagnosis. We compared the frequency and severity of respiratory exacerbation/AECOPD ascertained in this manner with the frequency and severity of respiratory exacerbation/AECOPD based on electronic health records (EHR). As a validation of the respiratory exacerbation/AECOPD construct, whether by self-report or by EHR confirmation, we also assessed airway inflammatory data available from subsets of study participants who had bronchoalveolar lavage (BAL) sampling.

## Methods

### Study design

**Fig. 1:**
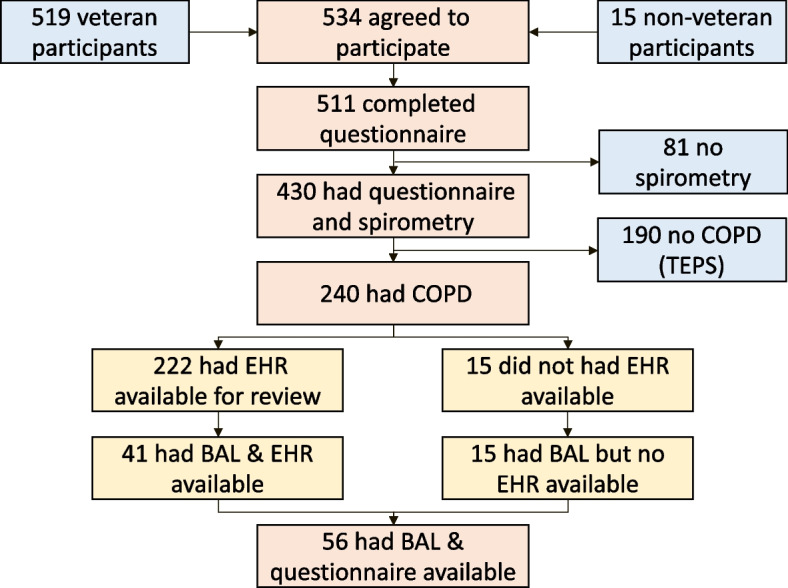
Participant flow through the study. Abbreviation: COPD = chronic obstructive pulmonary disease; TEPS = tobacco-exposed person with preserved spirometry; BAL = bronchoalveolar lavage; EHR = electronic health records

To investigate the clinical characteristics and inflammatory markers of acute respiratory exacerbations associated with smoking and/or COPD, we conducted a prospective observational study of current or former smokers with and without diagnosis of COPD at the San Francisco Veterans Affairs Health Care System (SFVAHCS). The participants, who could have been veterans or non-veterans, underwent baseline characterization through questionnaire administration and pulmonary function testing (PFT). The questionnaires included several standardized respiratory batteries as well as a COPD Respiratory Exacerbation Questionnaire previously developed by our group for quantification of respiratory symptom exacerbations associated with smoking and/or COPD in the preceding three years prior to participation in the study [16]. To determine the accuracy of self-reported episodes of respiratory exacerbation and AECOPD, we reviewed the EHR of the study participants, specifically focusing on the veterans who received care at SFVAHCS. To capture a greater number of patients with a history of respiratory exacerbation, we reviewed the EHR for the preceding three years (as opposed to one year), identifying any reports consistent with a respiratory exacerbation or an AECOPD event. We then determined the agreement between self-report and EHR-documentation of those events. In addition, a subset of participants were also invited to take part in a separate study that included bronchoscopy with bronchoalveolar lavage (BAL) to assess the airway inflammatory processes in COPD [16]. We examined the association of self-report and EHR-documented respiratory exacerbation and AECOPD events with the airway inflammation and cellularity data from the subset of the study participants who had BAL sampling. Our primary analysis was focused on the participants with spirometric COPD and their respiratory exacerbations (labeled as AECOPD) (Fig. 1). As a secondary analysis, we also examined the participants with history of smoking but no spirometric COPD (tobacco-exposed persons with preserved spirometry or TEPS) and their respiratory exacerbations (not labeled as AECOPD, as these participants did not have spirometric COPD).

### Study population

Participants were U.S. military veterans or non-veteran, current or former smokers, with and without history of COPD recruited through a study based at the SFVAHCS but not limited to persons receiving care there. Study inclusion criteria were age greater than 40 years and a history of 20 pack-years or more of tobacco use. We excluded potential participants with a concomitant diagnosis of asthma if asthma was clinically believed to be their primary respiratory disease. We also excluded potential participants with known lung cancer. To ensure that the participants’ testing and BAL sampling were not capturing an acute post-exacerbation inflammatory phase, those with a recent history of respiratory infection or exacerbation were only allowed to participate in the study a minimum of 6 weeks after their complete recovery.

The University of California San Francisco (UCSF) Institutional Review Board (IRB), the regulatory body associated with SFVAHCS, and the SFVAHCS Committee on Research and Development approved the study protocols. We obtained written IRB-approved informed consent and Health Insurance Portability and Accountability Act (HIPAA) from all study participants. All participants received monetary compensation for participating in the study.

### Other respiratory symptoms and health status

We assessed their respiratory and general health status symptoms using the modified Medical Research Council (mMRC) Dyspnea Scale [17], the Short Form 12-Item Health Survey (SF12) [18], COPD Assessment Test (CAT) [19], and a self-reported medical health questionnaire (SFVAHCS Pulmonary Research Medical Health Questionnaire) [16] to extract information regarding detailed use of tobacco and recreational drugs along with symptoms of dyspnea, cough, and sputum.

### Pulmonary function testing

Pulmonary function tests were performed using a model VyAir 229 CareFusion (VyAir Corp., Yorba Linda, CA) and nSpire body plethysmograph (nSpire Health Inc., Longmont, CO) in the seated position. This included measurement of the flow-volume curve and spirometry [20], lung volume by single breath dilution [21, 22] and plethysmography [23], airway resistance during panting at functional residual capacity (FRC) [24, 25], and single breath carbon monoxide diffusing capacity [26]. The pulmonary function studies was conducted based on guidelines provided by the American Thoracic Society (ATS) and European Respiratory Society (ERS) [27–32]. Categorization of participants was completed using the Global Initiative on Obstructive Lung Disease (GOLD) staging system [33] based on the results of spirometry performed before and after two inhalations of albuterol at a dose of 90 μg per inhalation.

### Assessment of self-report respiratory exacerbations and AECOPD

We determined the frequency and severity of respiratory exacerbation/AECOPD events using a COPD Exacerbation Questionnaire developed by our group (available at https://www.arjomandilab.ucsf.edu/copd-exacerbation-questionnaire), as described previously [16]. This questionnaire was established through a modification of the “Respiratory Disease Questionnaire” developed by the Genetic Epidemiology of COPD (COPDGene) study investigators for characterization of the respiratory symptoms and diseases of the research participants in the COPDGene cohort (available at https://www.copdgene.org/phase-1-study-documents.htm) [6]. The specific questions relevant for the assessment of the frequency and severity of respiratory exacerbations/AECOPD were extracted from that questionnaire and then revised to learn more about the frequency and severity of exacerbation in the preceding three years, instead of the preceding 12 months, before participation in our study. This approach to include a wider range of time allowed us to capture a greater number of patients with history of respiratory exacerbation/AECOPD, as recent cohort studies (COPDGene and SPIROMICS) have reported the retrospective incidence of AECOPD to be relatively low (24 to 30% for AECOPD of any severity and 11% for severe AECOPD requiring hospitalization [34].

The first part of the questionnaire elicited responses on emergency room visits and hospitalization, use of antibiotics, and the use of steroids for COPD. Furthermore, similar to COPDGene questionnaire, our modified version of the questionnaire inquires about the frequency of various levels of self-assessed exacerbation severity, including very mild (no special treatment required), mild (required only increasing home inhaler medications), moderate (required taking additional antibiotic or steroid medication kept at home), moderately severe (required consulting their doctor who prescribed them antibiotics and/or steroid treatment), severe (required admission to hospital), or very severe exacerbation (required admission to intensive care unit and/or intubation with mechanical ventilation). We calculated the frequencies of each of the six categories of exacerbation episodes and also combined them to dichotomize between very mild to mild vs. moderate to very severe.

### Assessment of health records-documented respiratory exacerbations and AECOPD

The veteran study participants who comprised most of those included almost exclusively obtained their health care through the Department of Veterans Affairs Healthcare Systems with a unified and connected electronic medical record platform. On the other hand, the subset of non-veteran study participants obtained their care through different non-VA healthcare systems with distinct and unconnected medical records platforms. Thus, the VA health records provide a better opportunity for more comprehensive capture of the participants’ exacerbation episodes through evaluation of medical records. Given the above, we focused our quantification of frequency and severity of respiratory exacerbation/AECOPD within the medical records to the veteran participants. Accordingly, Computerized Patient Record System (CPRS) records from SFVAHCS and other nationwide VA healthcare systems were examined by two independent reviewers according to an a priori protocol developed by our research team. The CPRS records were surveyed beginning three years before and up to the date on which the participants were enrolled and completed the AECOPD Questionnaire.

Based on the protocol, we interrogated SFVAHCS outpatient and inpatient records, which included outpatient visit notes from primary care providers (“medical practice”) and specialty clinic providers (“chest clinic”) and inpatient visit notes associated with SFVAHCS emergency department visits, hospitalization records, and discharge summaries. We searched for additional respiratory exacerbation/AECOPD episodes in the electronic health record using the search function, inputting the keywords “COPD” and “exacerbation” to find any potentially missed exacerbations. Records of other VA Healthcare Systems (outside SFVAHCS) outpatient and inpatient care were also reviewed if mentioned in the providers notes at SFVAHCS, through review of the Department of Veterans Affairs Joint Legacy Viewer (JLV). Outpatient and inpatient care at non-VA healthcare systems beyond what was available within the CPRS records was not evaluated.

For participants with COPD, the outpatient and inpatient visits were categorized to be due to AECOPD if exacerbation was documented as one of the diagnoses for the visits in the medical providers’ notes as detailed below. Documentation of moderate to very severe AECOPD was confirmed using the diagnoses documented in the medical records from the medical encounter (call to the clinic, clinic visit, urgent care visit, ED visit, or hospital stay) and the medications that were prescribed during and after that encounter (Supplemental Figure S1). For hospital admissions, both admission and discharge diagnoses were evaluated along with the medications prescribed during hospitalization and at discharge (Supplemental Figure S1). Care was taken to ensure the respiratory exacerbation episodes classified as AECOPD were true COPD exacerbation rather than respiratory problems due to other underlying comorbidities such as cardiovascular problems (myocardial infarction, congestive heart failure, etc.), pulmonary embolism, pure pneumonia without AECOPD, or other respiratory tract problems [12, 13]. If pneumonia and COPD exacerbation appeared as co-diagnoses, the exacerbation was recorded as an AECOPD only if steroids were also prescribed in addition to antibiotics. If pneumonia was the only diagnosis or no steroids were prescribed, the episode was not recorded as an exacerbation. If other respiratory or non-respiratory diagnoses that could require treatment with steroids (such as allergic or interstitial lung diseases) were cited as co-diagnoses, the exacerbation was recorded as an AECOPD only if antibiotics were also prescribed in addition to steroids. To distinguish new from slow-to resolve exacerbations or relapses, AECOPD episodes that were < 1 month apart were considered to be the same episode [35, 36]. Quantification of the respiratory exacerbation events in TEPS followed the same approach.

### Bronchoscopy and bronchoalveolar lavage (BAL) cell assessment

Bronchoscopy with BAL was performed in a subset composed of both veteran and non-veteran study participants (Fig. 1). Although all participants were invited to participate in the bronchoscopy part of the study, those with history of exacerbation were more actively recruited to capture a greater number of patients who had such events. Bronchoscopy was done within 1 to 4 weeks after participant enrollment in the study and completion of the questionnaires, as a matter of convenience for the participants and as was permitted based on the availability of the clinical bronchoscopy service. All visits including bronchoscopy visit were done when the participants perceived that they were at their baseline health status and in particular, a minimum of 6 weeks after any reported respiratory illnesses involving upper or lower airways.

The procedures of bronchoscopy and BAL have been previously discussed in detail [37]. Concisely, we established intravenous access, delivered supplemental oxygen, and anesthetized the upper airways using topical lidocaine. Intravenous fentanyl and midazolam were used for sedation as if deemed necessary for subject comfort. The bronchoscope was introduced through the mouth and vocal cords into the airways and finally reached into the right middle lobe. We performed lavage with two 60-ml aliquots (total of 120 mL) of pre-warmed 0.9% saline in each of medial and lateral segments of the lobe (total of 240 mL). The BAL was collected in a polyethylene tube and placed on ice transiently during transport to laboratory for appropriate processing. A small aliquot (1 ml) of the BAL was separated for counting of cells, and the remainder was immediately centrifuged at 4 °C and 180 g for 15 min to isolate cells from fluid for future studies.

We first counted the total number of cells from uncentrifuged aliquots of BAL using a hemocytometer followed by the differential cell counts from slides prepared using a cytocentrifuge, 25 g for 5 min, and stained with Diff-Quik (Dade Behring, Düdingen, Switzerland), as previously described [37]. Two hundred immune cells were counted by two independent observers at 100 × magnification using immersion oil; and we averaged the two counts for data analysis.

### Data analysis

We examined the distributions of participants’ characteristics and pulmonary function. We calculated percent predicted as well as upper and lower limits of normal (ULN and LLN) values for spirometric measures using Global Lung Function Initiative (GLI) [38]. We reported summary statistics of mean and standard deviation for continuous variables, and the number and percentage for binary and categorical variables.

Self-reported and EHR-documented history of exacerbations were quantified by number of incidents with respect to severity categories over the preceding three years from the date that the participant completed the AECOPD questionnaire. The quantification in either self-reported or EHR-documented approach consisted of calculation of the total number of respiratory exacerbation/AECOPD in each severity category (very mild to very severe) for each participant separately, and then summation of all the events to obtain a total count for each participant as well as the entire cohort. In addition, a combined count of self-reported and EHR-documented exacerbation events was generated by taking the highest number of exacerbation events by self-report *or* EHR documentation at each severity level, and then the summation of those across severity categories of interest.

Binary classification of occurrence of exacerbations with respect to each severity category were also generated, including binary classification of having any history of moderate to very severe exacerbation events in the preceding three years. The agreement between binary classification of self-reported and documented history of exacerbation events was measured by percent agreement and Cohen’s kappa [39].

To account for possible concerns about the unsuitability of the EHR to capture very mild and mild episodes of exacerbation, which by definition do not require healthcare system interaction and may not result in health record generation, the main analysis focused on the total number of moderate to very severe exacerbation.

In the subset of participants with BAL data, we estimated the association between the total number of moderate to very severe exacerbation events (by self-report, EHR documentation, or both combined) and each measure of airway inflammation using multivariable regression modeling with adjustment for covariates including age, sex, height, weight, and smoking status (current versus former smoking) and burden (pack-years of smoking). The parameter estimates of the associations were then compared by t-test using their standard errors computed from the corresponding regression models. Former smoking was defined as no smoking for ≥ 1 year, as the acute inflammatory effects of smoking (such as serum levels of tumor necrosis factor-α) have been shown to decline up to at least 12 months after smoking cessation [40]. In addition, we conducted a sensitivity analysis to examine the association between each measure of airway inflammation and time from the last m/s-AECOPD using linear regression modeling with adjustment for covariates.

For each regression model, the total number of participants, the parameter estimates, 95% confidence intervals (CI), and the *P* values from the regression modeling were reported. Data management, regression modeling, and visualization of regression results were done in R (version 4.2.1; R Foundation for Statistical Computing, Vienna, Austria).

## Results

### Participants characteristics

Beginning November 1, 2015 through April 22, 2022, we recruited 534 patients with a history of ≥ 20 pack-years of current or former smoking to participate in the study (Fig. 1). Among those, 511 completed AECOPD questionnaire data, from whom 430 had lung function measurements available. Among those, 59% (240/430) had spirometric COPD and 41% (190/430) had preserved spirometry (categorized as TEPS). Finally, a subset of 74 (56 with COPD and 18 without [TEPS]) underwent bronchoscopy with BAL. EHR evaluation was available for 487 (95%) of the 511 participants, out of whom 222 had COPD and 184 were TEPS.

Characteristics of the participants with spirometric COPD are shown in Table 1. Overall, the participants with COPD were mostly men (95%), were 69.3 ± 7.0 years of age, 42% current smokers, and had a 69.3 ± 39.5 (median [interquartile range] = 57.0 [44.0, 94.3]) pack-years of smoking history. Among the 222 participants who were assessed by both self-report and EHR document evaluation, a total of 323 episodes of self-reported AECOPD (212 m/s-AECOPD) and 146 episodes of EHR-documented AECOPD (133 m/s-AECOPD) were identified (Table 2).

**Table 1:** Characteristics of participants with spirometric COPD

Participants with COPD	All	Those with EHR available	Those with BAL sampling
No.	240	222	56
Age (years)	69.3 ± 7.0	69.8 ± 6.8	66.1 ± 6.0
Sex [Female n (%)]	13 (5.4%)	8 (3.6%)	5 (8.9%)
Height (cm)	176 ± 10	176 ± 10	175 ± 7
Weight (kg)	86.1 ± 21.8	86.3 ± 21.6	89.1 ± 24.5
BMI (kg/m^2^)	27.9 ± 8.4	28.0 ± 8.5	28.9 ± 7.0
Current Smoker [n (%)]	101 (42.1%)	94 (42.3%)	25 (44.6%)
Smoking history (pack-years)			
Mean ± standard deviation	69.3 ± 39.5	70.2 ± 37.5	60.0 ± 38.9
Median [interquartile range]	57.0 [44.0, 94.3]	58.8 [46.0, 96.0]	54.5 [39.4, 66.1]
FEV_1_ (% predicted)	68 ± 23	70 ± 23	53 ± 14
FVC (% predicted)	94 ± 36	95 ± 36	79 ± 16
FEV_1_/FVC (actual ratio)	0.56 ± 0.11	0.56 ± 0.11	0.52 ± 0.11
FEV_1_/FVC (% predicted)	73 ± 15	73 ± 15	67 ± 14
Spirometric COPD			
GOLD stage 1 [n (%)]	71 (29.6%)	70 (31.5%)	0 (0%)
GOLD stage 2 [n (%)]	122 (50.8%)	112 (50.5%)	35 (62.5%)
GOLD stage 3 [n (%)]	36 (15.0%)	32 (14.4%)	16 (28.6%)
GOLD stage 4 [n (%)]	10 (4.2%)	7 (3.2%)	5 (8.9%)
CAT	13.2 ± 8.29	13.2 ± 8.29	–
mMRC	1.15 ± 1.07	1.15 ± 1.07	–
SF12			
Physical component score	41.4 ± 5.6	41.4 ± 5.6	–
Mental component score	46.4 ± 5.1	46.4 ± 5.1	–

Demographics and lung function in participants with COPD. Data are presented as mean ± standard deviation or number of participants with positive value for the variable (n) and percentage of participants (%) out of the total number of participants. Reference equations: percent predicted of normal values of spirometry were calculated using Global Lung Function Initiative (GLI) [20]

*Abbreviations*: *BMI* Body mass index, *FEV*_*1*_ Forced expiratory volume in 1 s, *FVC* Forced vital capacity, *GOLD* Global Initiative on Obstructive Lung Disease, *CAT* COPD Assessment Test, *mMRC* modified Medical Research Council Dyspnea Scale, *SF12* Short Form-12

**Table 2:** The prevalence and the number of exacerbation episodes and their severity by self-report and EHR documentation among participants with COPD

Participants with COPD	All participants	Those with EHR available	Those with BAL sampling
**Self-reported AECOPD**	***n* = 240**	***n* = 222**	***n* = 56**
Total No. of subjects with any exacerbations [n (%)]	91 (37.9%)	75 (33.8%)	40 (71.4%)
Total No. of subjects with ≥ 1 per year m/s-AECOPD [n (%)]	30 (12.5%)	24 (10.8%)	10 (17.9%)
Total No. of subjects with ≥ 2 per year m/s-AECOPD (“frequent exacerbator”) [n (%)]	19 (7.9%)	15 (6.8%)	7 (12.5%)
Average No. of m/s-AECOPD per year in those with AECOPD (mean ± SD)	0.92 ± 1.19	0.94 ± 1.25	0.82 ± 1.21
Total No. of all exacerbations	392	323	182
Very mild	75	61	49
Mild	67	50	35
Moderate	73	62	31
Moderately severe	100	82	41
Severe	60	55	23
Very severe	17	13	3
Total No. of all moderate to very severe exacerbations	250	212	98
**EHR-documented AECOPD**		***n* = 222**	***n* = 41**
Total No. of subjects with any exacerbations [n (%)]	-	69 (31.1%)	22 (53.7%)
Total No. of subjects with ≥ 1 per year m/s-AECOPD [n (%)]	-	18 (8.1%)	6 (14.6%)
Total No. of subjects with ≥ 2 per year m/s-AECOPD (“frequent exacerbator”) [n (%)]	-	2 (0.9%)	1 (2.4%)
Average No. of m/s-AECOPD per year in those with AECOPD (mean ± SD)	-	0.64 ± 0.44	0.68 ± 0.45
Total No. of all exacerbations	-	146	49
Very mild	-	7	1
Mild	-	6	3
Moderate	-	1	1
Moderately severe	-	99	34
Severe	-	28	7
Very severe	-	5	3
Total No. of all moderate to very severe exacerbations	-	133	45

Details of AECOPD episodes by self-report and EHR documentation. Participants may have reported or had documentation of one or more AECOPD episodes of different severities. Data are presented as number of participants with positive value for the variable (n) and percentage of participants (%) out of the total number of participants. Total number of all reported AECOPD episodes and their severities are also reported

Corresponding data for the TEPS participants are shown in Supplemental Table S1 and Supplemental Table S2. Compared to those with COPD, fewer TEPS had respiratory exacerbations by either quantification methods. For example, 4% of TEPS versus 11% of those with COPD reported having had an average of one or more moderate to very severe exacerbation annually by self-report (*P* value from adjusted regression model = 0.002). Similarly, 1% of TEPS versus 8% of those with COPD had an average of one or more moderate to very severe exacerbations annually by their EHR-documentation (*P* value from adjusted regression model = 0.012) (Table 2 and Supplemental Table S2). However, among those with any history of exacerbation, the difference in the frequency of moderate to very severe exacerbation between TEPS and those with COPD did not reach statistical significance (Self-report: 0.68 ± 0.87 exacerbations per year in TEPS versus 0.94 ± 1.25 in those with COPD [*P* value from adjusted regression model = 0.391]; EHR-documented: 0.43 ± 0.27 exacerbations over three years in TEPS versus 0.64 ± 0.44 in those with COPD [*P* value from adjusted regression model = 0.225]).

### Self-report and EHR-identified AECOPD agreement

Among the 222 participants with COPD with available EHR data, 75 (34%) self-reported at least one episode of AECOPD in the three years preceding study. Among those, 57 (76%) had reported having at least one moderate to very severe AECOPD (m/s-AECOPD) in the same period of time, while 18 (24%) reported only very mild or mild exacerbation (Table 2). On review of the corresponding EHR data, 69 out of 487 (31%) were found to have any documented AECOPD over the same three-year time period. Among those, 66 (96%) had been documented to have had at least one m/s-AECOPD and 3 (4%) were documented to have had very mild or mild exacerbation (*P* < 0.001 for difference between self-report versus EHR documentation in the number of participants with only very mild or mild AECOPD). Overall, the total number of self-reported very mild and mild AECOPD across all of those with any history of exacerbation was significantly larger than the number that was discoverable in their EHR documentation (difference [95% CI] = 0.44 [0.22, 0.66]; *P* < 0.001).

The agreements between having had, or not having had, a self-reported and an EHR-documented m/s-AECOPD are shown in Table 3. Although the overall proportion of m/s-AECOPD by self-report (57/222; 26%) was similar to the proportion documented by EHR (66/222; 30%), there was substantial non-overlap (Cohen’s Kappa κ = 0.47 ± 0.06), which was consistent with only moderate agreement. We also calculated the agreement between self-report and EHR-documented m/s-AECOPD after excluding the 137 participants who did not have any history of m/s-AECOPD by either measure. Among the 85 remaining (all with history of m/s-AECOPD by one or another method), the kappa statistic was consistent with no agreement (κ = -0.36 ± 0.10). Among the subset with available EHR who underwent bronchoscopy (*n* = 41), having had, or not having had, a self-reported and an EHR-documented m/s-AECOPD also had a low Cohen’s Kappa agreement of 0.27 ± 0.15, which was consistent with only fair agreement (Table 4). The distribution of underreporting was relatively uniform across the three-year time period of assessment. For example, among the 10 participants with COPD who had severe to very severe AECOPD (hospitalizations or ICU stays) but did not self-report any, three had their AECOPD in the year before, three in 1 to 2 years before, and four in the 2 to 3 years before participation in the study.

**Table 3:** The contingency table for assessment of agreement between self-reported and EHR-documented moderate to very severe AECOPD (m/s-AECOPD) among the COPD subset with EHR evaluation

		**Self-reported m/s-AECOPD**		
		**No**	**Yes**	**Total**
**EHR-documented m/s-AECOPD**	**No.**	137	19	156
	**Yes**	28	38	66
	**Total**	165	57	222

Agreement between having or not having self-reported and EHR-documented moderate to very severe acute respiratory exacerbation episodes of COPD. There was substantial non-overlap (Cohen’s Kappa κ = 0.47 ± 0.07), consistent with only moderate agreement. Furthermore, when those without any history of m/s-AECOPD were excluded (*n* = 137), among the remaining 85 participants with history of at least one m/s-AECOPD by either self-report or EHR-documentation, there was no agreement (κ = -0.36 ± 0.10)

*Abbreviations: m/s-AECOPD* moderate to very severe acute exacerbation of chronic obstructive pulmonary disease, *EHR* Electronic health records

**Table 4:** The contingency table for assessment of agreement between self-reported and EHR-documented moderate to very severe AECOPD (m/s-AECOPD) among COPD subset with BAL data

		**Self-reported m/s-AECOPD**		
		**No**	**Yes**	**Total**
**EHR-documented m/s-AECOPD**	**No.**	14	6	20
	**Yes**	9	12	21
	**Total**	23	18	41

Agreement between having or not having self-reported and EHR-documented moderate to very severe acute respiratory exacerbation episodes of COPD. There was substantial non-overlap (Cohen’s Kappa κ = 0.27 ± 0.15), consistent with only moderate agreement. Furthermore, when those without any history of m/s-AECOPD were excluded (*n* = 14), among the remaining 27 participants with history of at least one m/s-AECOPD by either self-report or EHR-documentation, there was no agreement (κ = -0.36 ± 0.05)

*Abbreviations: m/s-AECOPD* moderate to very severe acute exacerbation of chronic obstructive pulmonary disease, *EHR* Electronic health records

Among the 184 TEPS participants with available EHR data, there was also a weak to moderate agreement between the number of participants with self-reported and EHR-documented respiratory exacerbations (Cohen’s Kappa κ = 0.38 ± 0.07) (Supplemental Table S3 and Supplemental Table S4).

### Association of AECOPD with airway inflammation

**Fig. 2:**
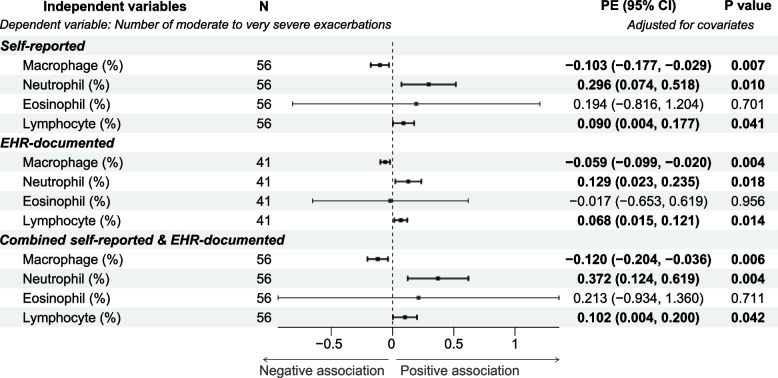
Associations between moderate to very severe AECOPD frequency and BAL inflammatory cells fractions. The associations of moderate to very severe AECOPD frequency with BAL inflammatory cells fractions were examined using linear regression modeling with adjustment for age, sex, height, weight, and smoking status and burden. The number of participants (N), the parameter estimates (PE), and the corresponding 95% confidence interval (CI) as well as *P* values are shown. The dot-and-whisker plots represent the PE and 95% CI. The PE, 95% CI, and dot-and-whisker plots for the statistically significant associations are shown in bold. Abbreviations: BAL: bronchoalveolar lavage; PE: parameter estimate; CI: confidence interval

**Fig. 3:**
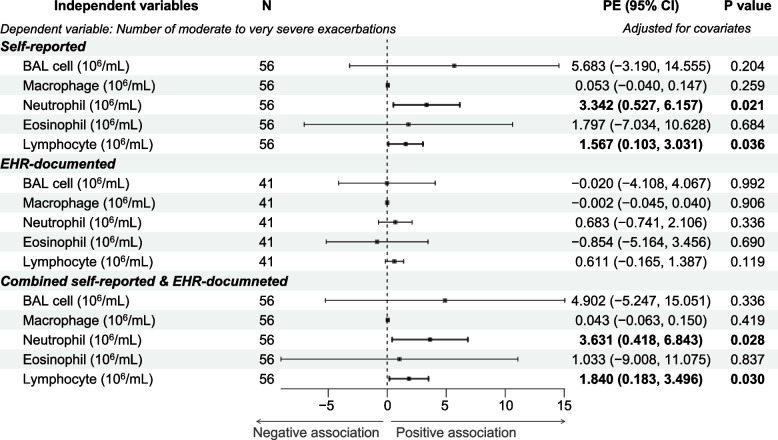
Associations between moderate to very severe AECOPD frequency and BAL inflammatory cells concentrations. The associations of moderate to very severe AECOPD frequency with BAL inflammatory cells concentrations were examined using linear regression modeling with adjustment for age, sex, height, weight, and smoking status and burden. The number of participants (N), the parameter estimates (PE), and the corresponding 95% confidence interval (CI) as well as *P* values are shown. The dot-and-whisker plots represent the PE and 95% CI. The PE, 95% CI, and dot-and-whisker plots for the statistically significant associations are shown in bold. Abbreviations: PE: parameter estimate; CI: confidence interval

In multivariable regression modeling in participants with COPD (*n* = 56) with adjustment for age, sex, height, weight, and smoking status (current vs. former) and smoking burden (pack-years), the number of self-reported m/s-AECOPD was significantly associated with both the percentage and the concentration of BAL neutrophils and lymphocytes (*P* values for all comparisons ≤ 0.041) (Figs. 2 and 3). The number of EHR-documented m/s-AECOPD was also significantly associated with the percentage of BAL neutrophils and lymphocytes (*P* values for all comparisons ≤ 0.018) (Figs. 2 and 3), but its associations with BAL cell concentrations were not significant. The combined number of self-reported and EHR-documented m/s-AECOPD was associated with the percentage and the concentration of BAL neutrophils and lymphocytes (*P* values for all comparisons ≤ 0.042) (Figs. 2 and 3).

The coefficients of association were nominally larger in the combined count model of m/s-AECOPD as opposed to those from the models relying on self-report or EHR-documentation only (Figs. 2 and 3), but the differences did not reach statistical significance with one exception. The coefficient (ß [95%CI]) for percent BAL neutrophils for combined model was significantly larger than that for EHR-documentation only model (0.372 [0.124–0.619] versus 0.068 [0.015–0.121], respectively; *P* = 0.035).

As a sensitivity analysis, we examined the effect of time from the last m/s-AECOPD, which was greater than 6 weeks, on BAL cell counts using linear regression modeling with adjustment for covariates. The analysis, which was only available for the subgroup of participants who had BAL and EHR-documented m/s-AECOPD, found no association between any of the BAL cell counts and the time from the last m/s-AECOPD (Supplemental Table S5). Furthermore, including the time from the last m/s-AECOPD had nominal effect on the main analysis of association of exacerbation frequency with BAL neutrophilia. However, inclusion of time elapsed since last m/s-AECOPD in the analysis led to attenuation of the association of exacerbation frequency with 95% confidence intervals that included the null (Supplemental Table S5).

## Discussion

The accurate identification and assessment of COPD exacerbations plays a pivotal role in understanding disease progression, optimizing patient management, and evaluating treatment efficacy [41]. Unfortunately, there is no validated diagnostic test or biomarker of exacerbations [42]. Hence, the determination of an exacerbation's diagnosis necessitates reliance on a clinical description encompassing the prevailing symptoms typically witnessed during such occurrences. In the present study, we used a self-assessment questionnaire to determine the frequency and severity of COPD exacerbation episodes as a means of capturing exacerbation events, with subsequent confirmation through cross-referencing with patients' medical records. This methodology aimed to explore the reliability and validity of patient-reported exacerbation data and its alignment with documented medical records. We found that there was moderate agreement between self-reported and EHR-identified AECOPD events suggesting that while both self-report and EHR methods capture many AECOPD events, there are notable discrepancies. Specifically, nearly half of the participants with EHR-identified moderate to very severe AECOPD events did not report any moderate to very severe exacerbations, and a similar proportion of participants with self-reported events did not have any moderate to very severe AECOPD recorded in their EHR (the nationwide Department of Veterans Affairs EHR). This finding highlights the limitations of relying solely on one data source for tracking AECOPD and emphasizes the importance of considering multiple data inputs when assessing exacerbation events. Despite the discrepancies between self-report and EHR-identified episodes of moderate to very severe AECOPD, the frequency of moderate to very severe AECOPD by either self-report or EHR-identified method was still associated with airway neutrophilia and lymphocytosis (markers associated with AECOPD risk) in the participants, remarkably when the sampling of the airway by BAL was performed in a stable condition with no recent (≥ 6 weeks) history of any respiratory exacerbation. This association implies that despite their discrepancies, self-report survey and EHR evaluation approaches are still suitable tools for quantification of AECOPD.

We further evaluated whether an approach that combined self-reported and EHR-documented AECOPD episodes could provide a quantification with larger effect size for airway inflammatory processes. We found that such an approach results in larger coefficients of association for AECOPD episode counts with the airway inflammatory cell counts, albeit not differing statistically from those based on self-report alone. It should be noted that our combined self-reported and EHR-documented approach was not able to truly capture all AECOPD events among the participants, as the matching of individual AECOPD episodes reported by participants with individual events identified in EHR documentation was not feasible due to unavailability of exact dates for the self-reported events. Instead, we simply took the highest count of AECOPD events in each severity category for each individual from either their self-reported or EHR-documented episodes and used it as combined quantification. This combined approach likely underestimates the real number of AECOPD events as well. Thus, it is possible that another combinatory approach that would provide a more accurate count of AECOPD events could provide a larger, statistically significant coefficient of association (effect size) with the AECOPD-relevant airway inflammatory markers.

We also conducted a similar analysis of self-reported and EHR-documented respiratory exacerbation among people with history of smoking but without COPD; that is, TEPS. As may be expected, compared to those with COPD, fewer participants with TEPS had respiratory exacerbation and among those who had exacerbation, there was a lower incidence of it. However, there were similar level of weak agreement between self-reported and EHR-documented respiratory exacerbations with nearly half of the EHR-documented ones not being reported by TEPS. There was no significant association between the frequency of respiratory exacerbations and BAL cell count in TEPS, but given the smaller number of exacerbations, the study may have been underpowered to find any association.

Because respiratory exacerbations in people with COPD or at risk for COPD (TEPS) are events with potential major clinical significance and prognostic relevance, their reliable characterization in frequency and severity is of great importance for both clinical care and research studies. Unless the patients are exclusively obtaining their care through a single payer healthcare system, quantification and classification of exacerbation episodes through medical records may be difficult if not impossible to ascertain. In fact, even in the case of single payer healthcare providers such as the Department of Veterans affairs, the patients may seek medical attention for the management of their exacerbation episodes through other healthcare systems depending on the urgency nature of their respiratory problems and/or convenience. Given the inadequacy of communication between different healthcare systems, examination of the medical records from any healthcare system for quantification of AECOPD may result in an underestimation of actual episodes. In our study, among those who received their care through the Department of Veterans Affairs, a single payer healthcare system, nearly half of the moderate to very severe exacerbations that were self-reported by the participants were not documented within their VA EHR. This discrepancy is consistent with anticipated patterns of health care seeking behavior, and may represent the actual AECOPD episodes for which the participants received care for outside the VA healthcare system.

As we show in this study, the frequency and severity of the AECOPD episodes can be obtained by surveying patients about the episodes they have experienced. The use of self-reported data is advantageous in that it may capture the actual frequency and severity of exacerbations, as patients and participants, as the primary observers of their health status, are more attuned to changes in their symptoms and their healthcare utilization. Self-report invariably introduces potential recall bias, including the tendency to better recall more severe events. However, we observed no concordance between self-reported and EHR-documented events when we limited the analysis to those with at least one such event by either metric. This suggests that misclassification may also be operative. Other studies in the past have reported some degree of under-reporting of respiratory exacerbation episodes among patients with COPD. In a prospective study of 421 patients with COPD, Langsetmo et al. showed that about one third of the patients under-reported worsening of their respiratory symptoms, not considering their symptoms to be an exacerbation episode [43]. However, these unreported episodes of worsening symptoms could not be considered to be of moderate to very severe severity that would require seeking medical attention. In another study by Seemungal et al., the authors reported under-reporting of moderate to severe AECOPD to comprise a small fraction (six out of 190 total episodes) of all AECOPD episodes among a selected group of 84 patients with severe COPD who obtained their routine care at an outpatient clinic [2]. In a three-year study of 409 COPD patients, Frei et al. reported the patients’ recall of AECOPD against a gold standard of medical record documentation to have a sensitivity of 84% and a specificity of 76%, although only 6% of those AECOPD episodes required hospitalization and thus could be considered to be severe or very severe [14].

Our study provides information about the incidence of AECOPD by self-report and EHR-documentation among a population of patients with history of relatively heavy smoking with or without spirometric COPD. Furthermore, our study provides additional information that despite their inaccuracy, self-report and EHR-documented AECOPD frequency were still significantly associated with airway inflammatory markers. In addition, our study also demonstrates that the frequency of moderate to very severe AECOPD computed by either self-report or EHR-identified to be associated with higher percentages and concentrations of neutrophils and lymphocytes in the airways, as measured by BAL sampling. Because the BAL sampling was carried out when the participants’ disease was stable with no recent history of AECOPD, the observed airway neutrophilia and lymphocytosis is suggestive of a link between airway inflammation and the occurrence of AECOPD and consistent with models proposing an airway pro-inflammatory profile in COPD patients who are prone to exacerbation [44–46]. Neutrophilic airway inflammation has been reported as a prominent feature of COPD that correlates with disease severity [47–51]. Nevertheless, airway neutrophilia as measured by sputum sampling has also been reported to be dissociated from the AECOPD occurrence rate [51]. The inconsistency between the findings of that report and the association of the airway neutrophilia with the frequency of AECOPD that we observed in our study could be related to the differences between the compartments of the proximal and/or distal airways that sputum and BAL procedures may sample [52]. Additionally, previous studies have reported an imbalance of airway lymphocyte subpopulations in patients with COPD and AECOPD [53–55]. We now show that the individuals who have had a higher rate of AECOPD in the past have a higher number of lymphocytes in their airways. While the observed association supports the use of quantification of AECOPD episodes by self-report in the context of this study, it may also further implicate airway lymphocytes as a culprit in AECOPD pathogenesis.

Interestingly, we did not find any significant association between the frequency of moderate to very severe AECOPD and BAL eosinophils, despite the previously reported association of these cells with COPD disease severity and exacerbation rate. *Airway* eosinophilia as measured by sputum has been shown to be weakly associated with AECOPD, while reports on the association of *blood*eosinophils with AECOPD have been mixed [56–59], with some suggesting an association while others not [59–61]. The inconsistency of the association of airway eosinophils with AECOPD between these reports may again reflect the differences between sputum and BAL procedure sampling [52].

Our study has several limitations that should be considered. First, we analyzed the agreement between self-reported and EHR-documented AECOPD episodes, which could amplify discrepancies between self-reported and EHR documentation of AECOPD episodes, because episodes less than moderate to very severe AECOPD by definition do not require healthcare system interaction and may not result in a medical record generation. However, to avoid any potential underreporting of AECOPD within medical records, we focused our main analysis on the total number of moderate to very severe AECOPD events, as those by definition require urgent care or emergency room visit and should result in a medical record or note generation, Second, the reliance on medical record documentation assumes the accuracy and completeness of clinical reporting. Variations in recording practices or potential under-diagnosing or over-diagnosing could impact the concordance observed between self-report and medical records. However, given this concern, we developed robust protocols to ascertain accurate determination of moderate to very severe AECOPD episodes from medical records by detailed review of the participants’ medical records. Third, our study's reliance on a predominantly Veteran study population may impact the generalizability of its findings, in particular because this was a predominantly male, heavy cigarette smoking study population. Cigarette smoking could potentially be a residual confounder even after accounting for it in multivariable modeling, although we do not have reason to believe that this is a major factor, and serving in military is associated with an increased risk of COPD even after adjustment for smoking status [62]. However, the majority of the US military veterans serve in the armed forces for 2 to 6 years, mainly during their youth, and then separate from the military and live their lives as other civilians, not unlike other countries worldwide with voluntary or mandatory military service [63]. The study of veterans provides a particular advantage by allowing access to a unified electronic health records from a single-payer health care system, providing a robust platform for analysis of self-report and EHR-documented AECOPD. Nonetheless, we acknowledge that generalization of our findings to other groups should take into account the potentially different characteristics of a Veteran cohort compared to others. Finally, the concerns about the invasive nature of bronchoscopy, as well as the higher subject payments that are customary for participation in it, may have subjected the bronchoscopy sub-study to selection bias. However, given the similar characteristics between those who participated in bronchoscopy and all participants, together with adjustment for covariates in the regression analysis, it is unlikely that our observations were resulted from such selection bias.

## Conclusions

In conclusion, we found that while there is some agreement between self-reported and EHR-identified incidence of AECOPD, a subset of AECOPD events is missed if any one method is used alone. An approach in which exacerbation history from self-report and medical record documentation are integrated offers an advantage to capture a more complete count of the exacerbation episodes. The careful cross-validation of self-reported occurrences with clinical data strengthens the credibility and accuracy of participant self-assessment and highlights the need for a more robust data collection approach. These findings hold implications for clinical practice as well as research strategies, emphasizing the importance of adopting a multifaceted approach to data collection to ensure accurate assessments. Remarkably, we also found that the incidence of moderate to very severe AECOPD by both self-report or EHR-identified methods to be associated with BAL neutrophilia and lymphocytosis in stable COPD, suggesting that despite its deficiencies, both self-reported history of moderate to very severe AECOPD has some biologic correlate. A combined approach to quantification of AECOPD by employing both self-report and EHR-documentation may be superior with a larger estimated effect than either method alone.

## Supplementary Information

Supplementary Material 1.

## Data Availability

The datasets generated and analyzed for this manuscript is available from the corresponding author on reasonable request.

## Data availability

The datasets generated and/or analyzed during the current study are available from the corresponding author on reasonable request.
