# Correction: Self‑report underestimates the frequency of the acute respiratory exacerbations of COPD but is associated with BAL neutrophilia and lymphocytosis: an observational study

**DOI:** 10.1186/s12890-025-04076-z

**Published:** 2026-01-29

**Authors:** Yorusaliem Abrham, Siyang Zeng, Wendy Lin, Colin Lo, Alexander Beckert, Laurel Evans, Michelle Dunn, Brian Giang, Krish Thakkar, Julian Roman, Paul D. Blanc, Mehrdad Arjomandi

**Affiliations:** 1 Medical Service, San Francisco Veterans Affairs Health Care System, San Francisco, CA USA; 2 Department of Medicine, University of California, San Francisco, CA USA; 3 Department of Biomedical Informatics and Medical Education, University of Washington, Seattle, USA; 4 Chicago College of Osteopathic Medicine, Midwestern University, Downers Grove, IL USA

**Correction: BMC Pulm Med 24**,** 433 (2024)**

**https://doi.org/10.1186/s12890-024-03239-8**

Following publication of the original article [1], an error was identified in the references.

The updated references are given below and the changes have been highlighted in **bold typeface**.

On page 5, Bronchoscopy and bronchoalveolar lavage (BAL) cell assessment sub-section:

We first counted the total number of cells from uncentrifuged aliquots of BAL using a hemocytometer followed by the differential cell counts from slides prepared using a cytocentrifuge, 25 g for 5 min, and stained with Diff-Quik (Dade Behring, Dudingen, Switzerland), as previously described [**37**].

On page 5, Data analysis sub-section, the current reference number [20] is incorrect. The appropriate reference was not included in the reference section. A new reference should be added as new reference [38], and current references [38]-[62] should be renumbered to [39]-[63], respectively:

We calculated percent predicted as well as upper and lower limits of normal (ULN and LLN) values for spirometric measures using Global Lung Function Initiative (GLI) [**38**].

A new reference entry should be added in the list:

**38. Hall GL**,** Filipow N**,** Ruppel G**,** Okitika T**,** Thompson B**,** Kirkby J**,** Steenbruggen I**,** Cooper BG**,** Stanojevic S; contributing GLI Network members. Official ERS technical standard: Global Lung Function Initiative reference values for static lung volumes in individuals of European ancestry. Eur Respir J. 2021 Mar 11;57(3):2,000,289. doi**: 10.1183/13993003.00289-2020.

The original article has been corrected.
