# Supplementary material for: Self-report underestimates the frequency of the acute respiratory exacerbations of COPD but is associated with BAL neutrophilia and lymphocytosis: an observational study
Source: BMC Pulm Med. 2024 Sep 2;24:433. doi: 10.1186/s12890-024-03239-8 (PMC11367895; doi:10.1186/s12890-024-03239-8)

SUPPLEMENTAL APPENDIX

Self-report underestimates the frequency of the acute respiratory exacerbations of COPD but is associated with BAL neutrophilia and lymphocytosis: an observational study

author list:

Yorusaliem Abrham, MD,[1,2] * Siyang Zeng, MS,[1,3] * Wendy Lin, BS,[4] Colin Lo, BS,[4] Alexander Beckert, RN,[2] Laurel Evans, BA,[1,2] Michelle Dunn, BS, BA,[1,2] Brian Giang, BA,[1,2] Krish Thakkar, BS,[1,2] Julian Roman, BA,[1,2] Paul D Blanc, MD, MSPH,[1,2] Mehrdad Arjomandi, MD[1,2]

AFFILIATIONS:

[1] Medical service, San Francisco Veterans Affairs Health Care System, San Francisco, California, USA

[2] Department of Medicine, University of California, San Francisco, California, USA

[3] Department of Biomedical Informatics and Medical Education, University of Washington, Seattle, USA

[4] Chicago College of Osteopathic Medicine, Midwestern University, Downers Grove, Illinois, USA

TABLE OF CONTENTS

Supplemental Table S1 2
Supplemental Table S2 4
Supplemental Table S3 6
Supplemental Table S4 7
Supplemental Table S5 8
Supplemental Figure S1 10

Supplemental Table S1

Characteristics of TEPS participants.

TEPS participants	All	Those with EHR available	Those with BAL sampling
No.	190	184	18
Age (years)	69.1±7.0	69.4±7.0	65.2±5.7
Sex [Female n (%)]	13 (6.8%)	11 (6.0%)	3 (16.7%)
Height (cm)	176±11	176±11	177±8
Weight (kg)	92.0±18.8	92.4±18.7	98.7±20.8
BMI (kg/m^2)	30.5±13.0	30.6±13.1	31.4±5.62
Current Smoker [n (%)]	74 (38.9%)	69 (37.5%)	9 (50.0%)
Smoking history (pack-years)			
Mean ± standard deviation	58.0±42.0	58.6±42.5	42.1±16.0
Median [interquartile range]	49.0 [32.0, 73.5]	50.0 [32.0, 76.0]	40.6 [32.8, 48.0]
FEV_1 (% predicted)	97±51	97±52	86±17
FVC (% predicted)	97±58	97±59	82±16
FEV_1/FVC (actual ratio)	0.77±0.05	0.77±0.05	0.80±0.04
FEV_1/FVC (% predicted)	100±7	100±7	104±6
CAT	9.56±6.68	9.56±6.68	7.13±3.40
mMRC	0.88±1.00	0.88±1.00	0.63±0.74
SF12			
Physical component score	42.4±4.5	42.4±4.5	44.3±5.0
Mental component score	45.4±4.9	45.4±4.9	45.0±5.4

Footnote: Demographics and lung function of TEPS participants. Data are presented as mean±standard deviation or number of participants with positive value for the variable (n) and

percentage of participants (%) out of the total number of participants. Reference equations: percent predicted of normal values of spirometry were calculated using Global Lung Function Initiative (GLI).[20] Abbreviations- BMI: body mass index; FEV_1: forced expiratory volume in 1 second; FVC: forced vital capacity; GOLD: Global Initiative on Obstructive Lung Disease; CAT: COPD Assessment Test, mMRC: modified Medical Research Council Dyspnea Scale; SF12: Short Form-12.

Supplemental Table S2

The prevalence and the number of respiratory exacerbation episodes and their severity by self-report and EHR documentation in TEPS participants.

TEPS participants	All	Those with EHR available	Those with BAL sampling
**Self-reported respiratory exacerbation**	**N=190**	**N=184**	**N=18**
Total No. of subjects with any exacerbations [n (%)]	25 (13.2%)	23 (12.5%)	3 (16.7%)
Total No. of subjects with ≥1 m/s-respiratory exacerbation per year [n (%)]	8 (4.2%)	7 (3.8%)	1 (5.6%)
Total No. of subjects with ≥2 m/s-respiratory exacerbation per year [n (%)]	4 (2.1%)	4 (2.1%)	4 (2.1%)
Average No. of m/s-respiratory exacerbation per year in those with respiratory exacerbation (mean ± SD)	0.72±0.88	0.68±0.87	0.78±1.07
Total No. of all exacerbations	95	80	14
Very mild	19	17	1
Mild	22	16	6
Moderate	17	13	3
Moderately severe	28	25	4
Severe	9	9	0
Very severe	0	0	0
Total No. of all moderate to very severe exacerbations	54	47	7
**EHR-documented respiratory exacerbation**		**N=184**	**N=13**

Total No. of subjects with any exacerbations [n (%)]	-	10 (5.4%)	1 (7.7%)
Total No. of subjects with ≥1 m/s-respiratory exacerbation per year [n (%)]	-	1 (0.5%)	0 (0%)
Total No. of subjects with ≥2 m/s-respiratory exacerbation per year [n (%)]	-	0 (0%)	0 (0%)
Average No. of m/s-respiratory exacerbation per year in those with respiratory exacerbation (mean ± SD)	-	0.433±0.274	0
Total No. of all exacerbations	-	14	1
Very mild	-	0	0
Mild	-	1	0
Moderate	-	0	0
Moderately severe	-	12	1
Severe	-	1	0
Very severe	-	0	0
Total No. of all moderate to very severe exacerbations	-	13	1

Footnote: Details of respiratory exacerbation episodes by self-report and EHR documentation. Participants may have reported or had documentation of one or more respiratory exacerbation episodes of different severities. Data are presented as number of participants with positive value for the variable (n) and percentage of participants (%) out of the total number of participants. Total number of all reported respiratory exacerbation episodes and their severities are also reported.

Supplemental Table S3

The contingency table for assessment of agreement between self-reported and EHR-documented moderate to very severe respiratory exacerbation among the TEPS subset with EHR evaluation.

		Self-reported m/s-respiratory exacerbation		
		**No**	**Yes**	**Total**
**EHR-documented m/s-respiratory exacerbation**	**No**	165	10	175
	**Yes**	4	5	9
	**Total**	169	15	184

Footnote: Agreement between having or not having self-reported and EHR-documented moderate to very severe (m/s-) respiratory exacerbation episodes among TEPS participants. There was substantial non-overlap (Cohen's Kappa κ=0.38±0.07), consistent with only moderate agreement. Furthermore, when those without any history of m/s-respiratory exacerbation were excluded (n=165), among the remaining 19 participants with history of at least one m/s-respiratory exacerbation by either self-report or EHR-documentation, there was no agreement (κ=-0.43±0.18). Abbreviations: m/s-respiratory exacerbation: moderate to very severe acute respiratory exacerbation; EHR: electronic health records.

Supplemental Table S4

The contingency table for assessment of agreement between self-reported and EHR-documented moderate to very severe respiratory exacerbation among TEPS subset with BAL data.

		Self-reported m/s-respiratory exacerbation		
EHR-documented m/s-respiratory exacerbation		**No**	**Yes**	**Total**
	**No**	11	1	12
	**Yes**	1	0	1
	**Total**	12	1	13

Footnote: Agreement between having or not having self-reported and EHR-documented moderate to very severe (m/s-) respiratory exacerbation episodes among the subset of TEPS participants with BAL data. There was substantial non-overlap (Cohen's Kappa κ=-0.08±0.28), consistent with only moderate agreement. Furthermore, when those without any history of m/s-respiratory exacerbation were excluded (n=11), among the remaining 2 participants with history of at least one m/s-respiratory exacerbation by either self-report or EHR-documentation, there was no agreement (κ=-1.00±0.70). Abbreviations: m/s-respiratory exacerbation: moderate to very severe acute respiratory exacerbation; EHR: electronic health records.

Supplemental Table S5

Effect of time from the last m/s-AECOPD on BAL cell counts and its association with exacerbation frequency.

	**Approach 1**	**Independent variable: Time from last m/s-AECOPD (days)**			
**N**	**Dependent variable in each model**	**PE**	**95% confidence interval**		**P value**
			**Lower bound**	**Upper bound**	
22	BAL cell (10^6/mL)	-0.0001	-0.0003	0.0001	0.39
	Macrophage (10^6/mL)	-0.0088	-0.0314	0.0137	0.41
	Neutrophil (10^6/mL)	-0.0002	-0.0008	0.0004	0.45
	Eosinophil (10^6/mL)	<0.0001	-0.0001	0.0001	0.76
	Lymphocyte (10^6/mL)	-0.0006	-0.0018	0.0006	0.28
	Macrophage (%)	0.0131	-0.0074	0.0335	0.19
	Neutrophil (%)	-0.0026	-0.0101	0.0050	0.48
	Eosinophil (%)	0.0004	-0.0008	0.0017	0.49
	Lymphocyte (%)	-0.0102	-0.0270	0.0067	0.21
	**Approach 2**	**Dependent variable: No. of m/s-AECOPD**			
**N**	**Independent variable in each model**	**PE**	**95% confidence interval**		**P value**
			**Lower bound**	**Upper bound**	
22	BAL cells (10^6/mL)	1.3904	-3.9715	6.7523	0.58
	Macrophage (10^6/mL)	0.0133	-0.0426	0.0693	0.61
	**Neutrophil (10^6/mL)**	**2.0590**	**0.1171**	**4.0010**	**0.03**
	Eosinophil (10^6/mL)	3.9777	-5.7957	13.7511	0.39
	Lymphocyte (10^6/mL)	0.1262	-0.9167	1.1691	0.79
	Macrophage (%)	-0.0326	-0.0918	0.0266	0.25
	Neutrophil (%)	0.1267	-0.0232	0.2766	0.09
	Eosinophil (%)	0.1311	-0.8763	1.1384	0.78
	Lymphocyte (%)	0.0213	-0.0531	0.0958	0.54
	**Approach 3**	**Dependent variable: No. of m/s-AECOPD**			
**N**	**Independent variable in each model**	**PE**	**95% confidence interval**		**P value**
			**Lower bound**	**Upper bound**	
41	BAL cells (10^6/mL)	0.7220	-2.4337	3.8777	0.64
	Macrophage (10^6/mL)	0.0065	-0.0266	0.0396	0.69
	Neutrophil (10^6/mL)	1.0344	-0.0281	2.0969	0.05
	Eosinophil (10^6/mL)	0.5034	-2.7445	3.7514	0.75
	Lymphocyte (10^6/mL)	0.1677	-0.4682	0.8036	0.59
	Macrophage (%)	-0.0305	-0.0647	0.0038	0.07
	**Neutrophil (%)**	**0.0881**	**0.0051**	**0.1710**	**0.03**
	Eosinophil (%)	0.0003	-0.4721	0.4726	0.99
	Lymphocyte (%)	0.0269	-0.0190	0.0728	0.24

Footnote: To determine whether time from the last m/s-AECOPD would affect BAL cell counts, we performed linear regression modeling with adjustment for covariates (i.e., age, sex, height, weight, and smoking status and burden) using three approaches: (1) examining the association between BAL cell counts and time from the last m/s-AECOPD in days as a continuous variable; (2) examining the association between number of m/s-AECOPD and BAL cell counts with inclusion of time from the last m/s-AECOPD in days as an additional continuous variable in the regression model; and (3) examining the association between number of m/s-AECOPD and BAL cell counts with inclusion of time from the last m/s-AECOPD as an additional categorical variable (i.e., within 1 year, 1 to 2 year, 2 to 3 years, and over 3 years) in the regression model. The number of participants (N), the parameter estimates (PE), and the corresponding 95% confidence interval (CI) as well as P values are shown. Abbreviations: m/s-AECOPD: moderate to very severe acute respiratory exacerbation of COPD; BAL: bronchoalveolar lavage; PE: parameter estimate; CI: confidence interval.

Supplemental Figure S1

Determination criteria of acute exacerbation of chronic obstructive pulmonary disease (AECOPD) episodes from the health records. For patients to be diagnosed to have had an AECOPD, they must have had one of the following criteria: (1) a discharge diagnosis of AECOPD; (2) an admission diagnosis of AECOPD plus inpatient and/or outpatient prescription of systemic steroids; (3) other admission or discharge respiratory diagnosis besides AECOPD but also inpatient and/or outpatient prescription of systemic corticosteroid and antibiotics. Determination of respiratory exacerbation episodes for tobacco-exposed persons with preserved spirometry followed similar criteria.

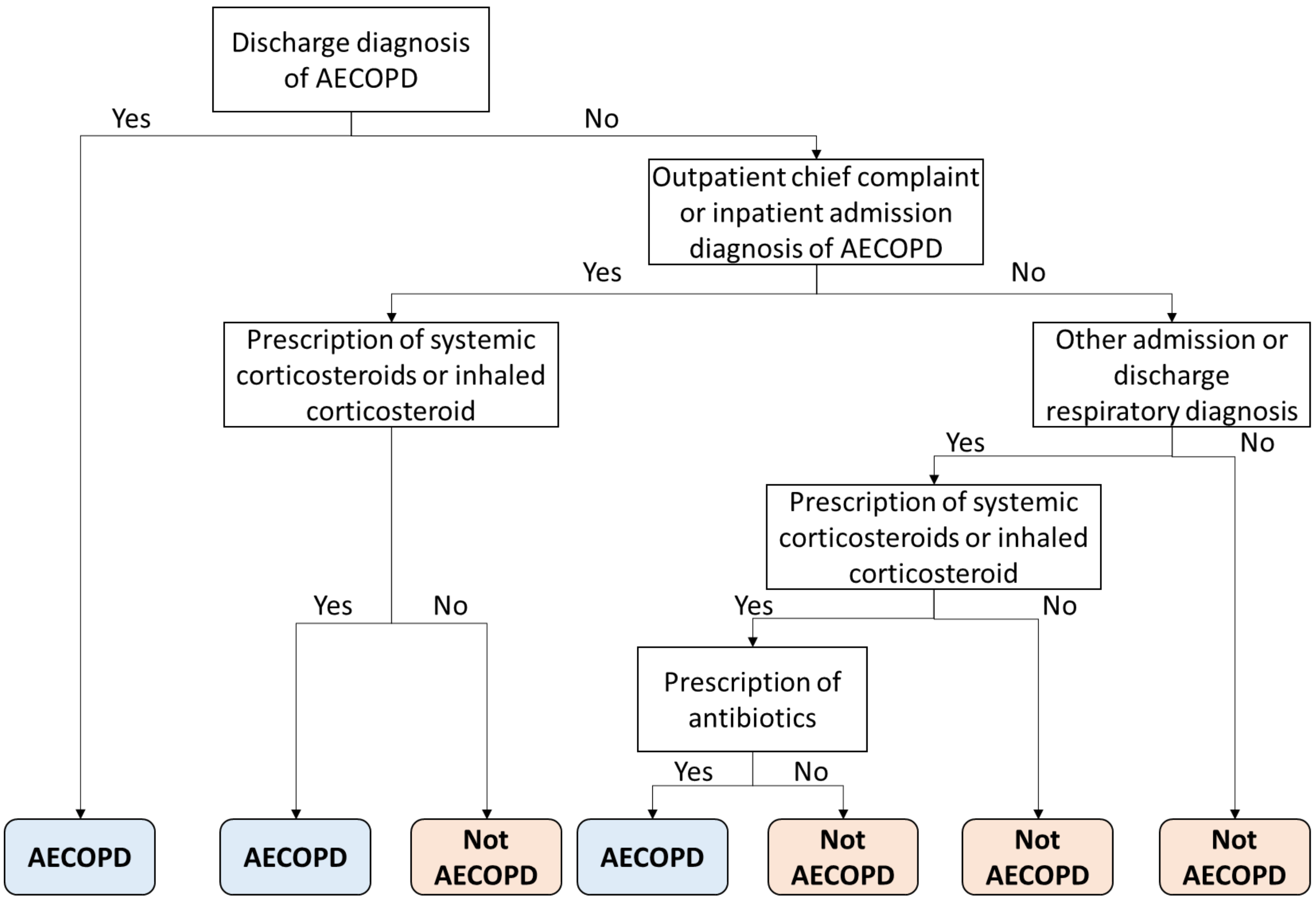

Supplement: Supplementary file 1 — Supplementary Material 1. [file 12890_2024_3239_MOESM1_ESM.pdf]
